# Public and social environment changes and caesarean section delivery choice in Japan

**DOI:** 10.1186/s13104-018-3746-2

**Published:** 2018-09-03

**Authors:** Michio Yuda

**Affiliations:** Graduate School of Economics and Management, Tohoku University, 27-1 Kawauchi, Aoba-Ward, Sendai, Miyagi 9808576 Japan

**Keywords:** Cesarean section delivery, Agency problem, Defensive medicine, Panel data, Japan

## Abstract

**Objective:**

As in many other countries, the ratio of caesarean section (c-section) delivery to total births in Japan is rising steadily, while the total number of deliveries is decreasing. Although c-sections can effectively prevent maternal and perinatal mortality and morbidity when medically justified, it is uncertain how medically unnecessary c-sections affect the short-, middle-, and long-term postnatal effects on the mother and child. As there are no empirical studies on c-section choice for Japan, this study uses individual medical facility panel data from 1999 to 2014 to comprehensively examine the effects of recent public and social environment changes on c-section delivery choice.

**Results:**

The empirical results from our fixed effect model show that c-section delivery and its ratio are higher in public hospitals, in relatively large clinics, and in clinics opening on holidays. In addition, increases in the lump-sum birth allowance and the number of medical malpractice lawsuits also increase the number of c-section delivery.

## Introduction

**Fig. 1:**
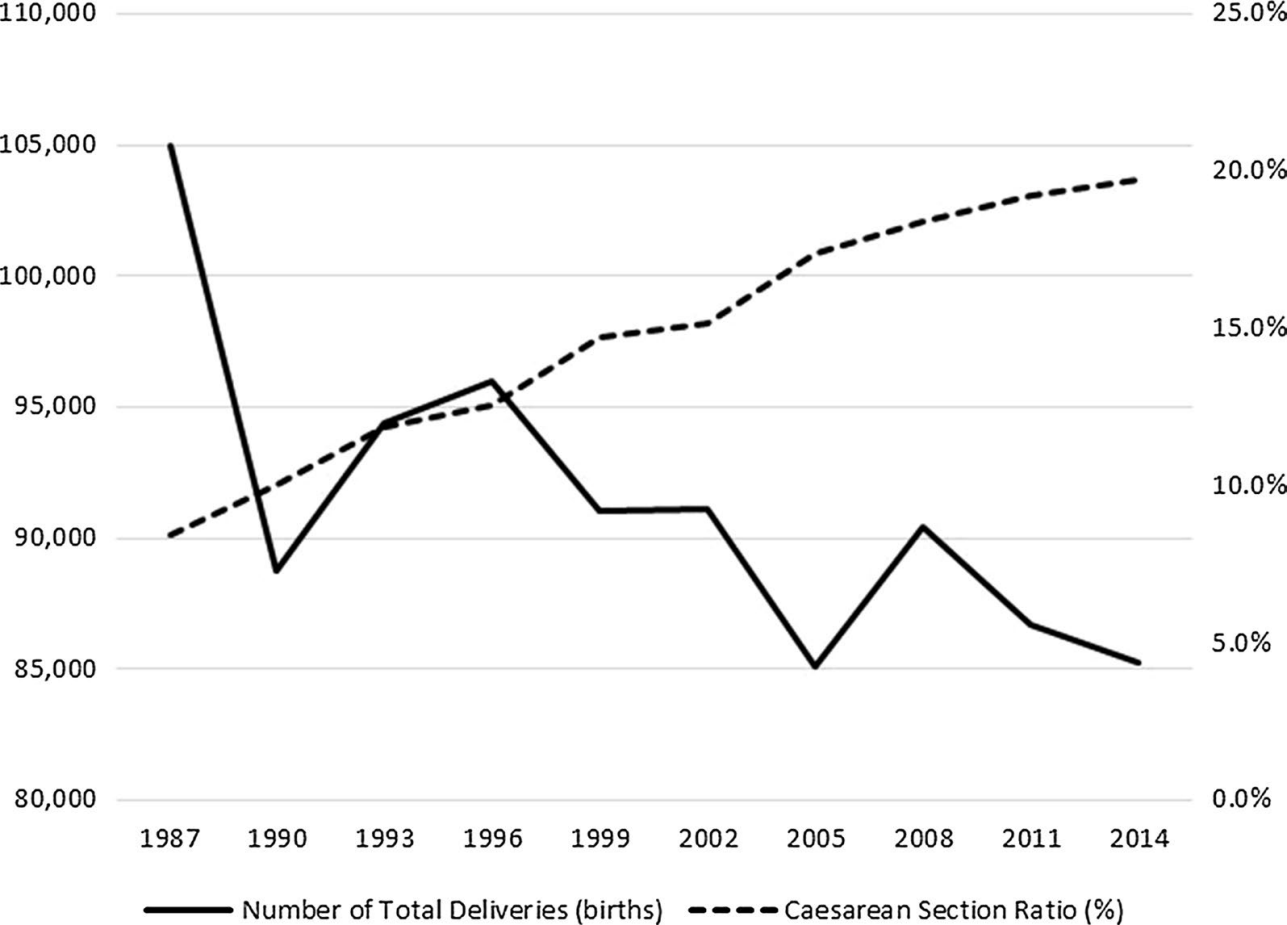
Total births and c-section ratio. Source: The Ministry of Health, Labour, and Welfare, the *Survey of Medical Institutions* in 2014

The World Health Organization [1] reports that caesarean section (c-section) delivery has increased in developed and developing countries since the 1980s. In Japan, the Ministry of Health, Labour and Welfare (MHLW) [2] reports that the ratio of c-section delivery to total births is rising steadily, while the total number of deliveries is decreasing (Fig. 1). Although c-sections can effectively prevent maternal and perinatal mortality and morbidity when medically justified, it is uncertain how medically unnecessary c-sections affect the short-, middle-, and long-term postnatal effects on the mother and child. Several empirical studies in health economics find that obstetricians and gynecologists choose c-sections for medical reasons, such as late childbearing and mother and infant health conditions [3, 4]. Other empirical health economics studies find that physicians choose c-sections because of their financial incentive [5], defensive medicine [6–9], public parenting support programs [10], local characteristics [11], and attributes of the medical facility and physicians [12–18].

In this study, I comprehensively estimate how public and social environment changes over time affect c-section delivery choice. In Japan, because vaginal deliveries are not covered by universal public health insurance benefits whereas c-sections are covered, households’ actual co-payments of c-sections are only slightly larger than those for a vaginal delivery. However, obstetricians and gynecologists may choose c-sections to reduce the medical risk to pregnant women and infants, and also because it is more profitable for them.^1^ However, there are no empirical studies investigating such incentives for Japan, as far as I know. In addition, because Norberg and Pantano [19] find that c-section delivery significantly decreases subsequent fertility, it is important for Japan’s fertility crisis to investigate which public and social factors significantly affect physicians’ c-section choice.

## Main text

### Data

The main data used in this study is the *Survey of Medical Institutions* (*Iryo Shisetsu Chosa*) from 1999 to 2014. This is the panel survey conducted by the Japanese MHLW every 3 years to obtain basic information about all medical facilities open at the time of the survey in Japan and their clinical functions for health-care policy-making. The study sample consisted of 4867 medical facilities (1958 hospitals and 2909 clinics^2^) with obstetrics and gynecology departments that had more than one childbirth during the study period. Table 1 summarizes the descriptive statistics of the main variables. As the characteristics of the hospitals and clinics differ considerably, I used data from all facilities in our empirical analysis.

**Table 1:** Descriptive statistics

Sample	Hospital		Clinic	
Variables	Mean	Std. dev	Mean	Std. dev
Endogenous variables				
Number of C-section deliveries	6.2	8.7	2.3	3.3
C-section ratio	0.2	0.2	0.1	0.1
Medical facility characteristics				
Public facility	0.6	0.5	0.0	0.1
Number of physicians	68.5	103.7	1.4	0.8
Number of sickbeds	331.7	238.5	11.9	5.7
Opening outside hours on weekdays	0.1	0.3	0.3	0.4
Opening on holidays	0.4	0.5	1.0	0.2
Aggregated variables				
Women’s average age for first marriage^a^	28.0	1.0	28.0	1.0
Child mortality rate^a^	0.7	0.2	0.7	0.2
Obstetricians and gynecologists density^b^	41.8	6.6	41.3	6.7
The youth population (thousand people)^c^	602.5	444.2	608.0	428.1
The amount of lump-sum birth allowance (10 thousand yen, 2010 price)^d^	34.4	6.1	34.2	6.1
Prefectural real GDP (billion yen, 2010 price)^e^	2.1	2.5	2.0	2.3
The number of yearly medical malpractice lawsuits (previous year)^f^	895.7	122.0	897.7	122.6
Number of observations (medical malpractice lawsuits)	9874 (8156)		13,631 (11,311)	
Number of facilities (medical malpractice lawsuits)	1958 (1882)		2909 (2814)	

^a^*Vital Statistics*, Ministry of Health, Labour, and Welfare; ^b^ *Survey of Physicians, Dentists and Pharmacists*, Ministry of Health, Labour, and Welfare; ^c^ *Population Census* and *Population Estimates*, Statistics Bureau, Ministry of Internal Affairs and Communications; ^d^ Ministry of Health, Labour, and Welfare [21]; ^e^ *Prefectural Economic Accounting*, Cabinet Office; and ^f^ *The Number of Medical*-*Malpractice Lawsuits by the Department*, Supreme Court of Japan

Regarding the hospital sample, the mean of c-section delivery is 6.2 births, with the annual mean increasing from 5.2 in 1999 to 7.3 in 2014. The ratio of c-section delivery to total deliveries for hospitals with more than one delivery is 17.6%, with the annual mean also increasing from 17.4% in 1999 to 25.0% in 2014. As for facility attributes, about half of the hospitals are public. The mean number of physicians (converted to number of full-time posts) is 68.5 persons and that of sickbeds is 331.7 beds. In addition, 7.8% and 37.8% of hospitals are open outside hours on weekdays and on holidays, respectively. However, the mean of c-section delivery at clinics is 2.3 births, with the annual mean increasing from 2.0 in 1999 to 2.6 in 2014. The c-section ratio for clinics with more than one delivery is 9.1%, with the annual mean increasing slightly from 11.4% in 1999 to 13.6% in 2014. As for facility attributes, almost all of the clinics are private. The mean number of full-time converted physicians is 1.4 persons and that of sickbeds is 11.9 beds. In addition, 28.2% and 95.1% of clinics are open outside hours on weekdays and on holidays, respectively.

### Methods

I estimate the following empirical equation with facility fixed effects to comprehensively examine the effect of various public and social environment changes on c-section choice.

$$y_{it} = \beta_{0} + {\varvec{\upbeta}}_{{\mathbf{x}}} {\mathbf{x}}_{{{\mathbf{it}}}} + {\varvec{\upbeta}}_{{\mathbf{z}}} {\mathbf{z}}_{{{\mathbf{it}}}} + \lambda_{i} + u_{it} .$$

The dependent variable *y*_*it*_ is the number of c-section deliveries or the c-section ratio of the medical facility *i* in year *t*. **x** is a vector of the facilities’ characteristics, which include the number of physicians and of sickbeds and dummy variables of public medical facility, opening outside hours on weekdays, and opening on holidays. The number of physicians and of sickbeds are proxies for the scale of the facility. According to the MHLW’s report, facility profit—medical practice revenue minus its costs—is generally negative for public medical facilities but positive for nonpublic ones. Therefore, the difference in organizational form may affect c-section choice. **z** contains aggregated prefectural variables^3^ reflecting the yearly and geographical variations in social environments, such as women’s average age at first marriage, child (aged 5 and under) mortality rate, density of obstetricians and gynecologists, youth population, amount of lump-sum birth allowance adjusted to 2010 prices, prefectural real gross domestic product (GDP), and number of yearly medical malpractice lawsuits.

As the *Survey of Medical Institutions* does not contain any information on the mothers and infants, the women’s average age at first marriage and child mortality rate are employed as proxies for their health risk. The definitions of the density of obstetricians and gynecologists and youth population are the same as those in Gruber and Owings [5]. The density of obstetricians and gynecologists is the number of them per 100,000 females aged from 15 to 49, which is the proxy for physicians’ financial incentive because of the competitive environment. The youth population is the number of people under 15 years of age, which is the proxy for the reduced birthrate. The lump-sum birth allowance is one of the public health insurance benefits paid in cash at the time of childbirth. The benefit was 300,000 yen in 1999 and increased to 420,000 yen in 2009 as an attempt by the government to reverse the declining birthrate. However, this increase in the benefit is similar to the increase in the average delivery costs over the same period. The recent increase in the delivery costs may be caused by an increase in the number of c-section deliveries in response to overall medical fee reductions in 2002 and 2006, and to its zero reform in 2004 that negatively affect medical facility’s financial condition. This supply-side response is regarded as an agency problem in the health-care market, i.e., supplier-induced demand (McGuire [20]). Real prefecture GDP is a proxy for yearly fixed effect, such as the business cycle. Medical malpractice lawsuits represent the number of new medical malpractice lawsuits in the previous year. This is a proxy for defensive medicine, because physicians may choose c-sections to reduce or avoid intranatal risks (defensive medicine). In addition, λ is the individual medical facility’s fixed effect and *u* is an error term.

### Results

Table 2 reports the estimation results. For the hospital sample, the coefficients of the public facility dummy variable are significantly positive. These results suggest that the number of c-sections in public hospitals is 56% to 80% larger and the c-section rate is 5.3% larger than other hospitals. Because hospital physicians have little financial incentive (see Footnote 1), relatively high-risk pregnant women deliver their children in public hospitals. In addition, the number of sickbeds also increases the c-section ratio, but its effect is small. As for the effects of the environment variables, youth population significantly increases both the number of c-sections and the c-section ratio but those effects are small. In addition, an increase in the amount of the lump-sum birth allowance also increases both the number of c-sections and the c-section ratio, which indicates that an agency problem exists. Moreover, an increase in women’s average age at first marriage increases c-section delivery but decreases the c-section ratio. In addition, an increase in the density of obstetricians and gynecologists significantly decreases c-section delivery, which indicates that little evidence that a financial incentive for physicians exists. Moreover, an increase in medical malpractice lawsuits significantly increases the c-section ratio, but its effect is also small, which indicates that there is little evidence of defensive medicine in Japan.

**Table 2:** Empirical results on c-section choices

Sample	Hospital				Clinic			
Dependent variable	C-section deliveries		C-section ratio		C-section deliveries		C-section ratio	
Medical facility characteristics								
Public facility	1.562^b^	1.759^b^	0.053^c^	0.034	− 0.346	− 0.075	− 0.028	− 0.027^b^
	(0.739)	(0.781)	(0.028)	(0.026)	(0.367)	(0.362)	(0.017)	(0.014)
Number of physicians	0.009	0.005	0.000	0.000	0.500^a^	0.558^a^	0.006^a^	0.009^a^
	(0.006)	(0.006)	(0.000)	(0.000)	(0.067)	(0.072)	(0.002)	(0.003)
Number of sickbeds	0.002	0.002	0.000^c^	0.000	0.103^a^	0.091^a^	0.005^a^	0.005^a^
	(0.004)	(0.005)	(0.000)	(0.000)	(0.010)	(0.011)	(0.001)	(0.001)
Opening outside hours on weekdays	0.512	0.660	0.005	0.008	0.107	0.250	− 0.001	0.002
	(0.333)	(0.506)	(0.011)	(0.010)	(0.148)	(0.172)	(0.005)	(0.006)
Opening on holidays	0.091	0.412	− 0.007	0.006	1.426^a^	1.364^a^	0.075^a^	0.072^a^
	(0.188)	(0.337)	(0.006)	(0.008)	(0.119)	(0.145)	(0.006)	(0.007)
Aggregated variables								
Women’s average age for first marriage	0.334^b^	− 0.030	− 0.028^a^	− 0.026^b^	0.144^b^	− 0.249	− 0.002	− 0.013
	(0.125)	(0.467)	(0.006)	(0.012)	(0.071)	(0.172)	(0.005)	(0.009)
Child mortality rate	− 0.002	0.006	0.010	0.000	0.078	0.034	0.002	− 0.003
	(0.240)	(0.226)	(0.008)	(0.005)	(0.098)	(0.087)	(0.005)	(0.005)
Obstetricians and gynecologists density	− 0.068^c^	− 0.063	0.000	0.001	− 0.019^c^	− 0.006	0.000	0.001
	(0.039)	(0.046)	(0.001)	(0.001)	(0.012)	(0.012)	(0.001)	(0.001)
The youth population	0.007^a^	0.010^a^	0.000	0.000^b^	0.004^a^	0.005^a^	0.000^a^	0.000^a^
	(0.002)	(0.002)	(0.000)	(0.000)	(0.001)	(0.001)	(0.000)	(0.000)
The amount of lump-sum birth allowance	0.034	0.099^c^	0.002^b^	0.002	0.006	0.066^a^	0.000	0.001
	(0.012)	(0.055)	(0.001)	(0.002)	(0.009)	(0.023)	(0.001)	(0.001)
Prefectural real GDP	1.865^a^	1.158^b^	0.045^c^	0.029^b^	0.080	− 0.178	0.001	0.001
	(0.499)	(0.476)	(0.026)	(0.014)	(0.270)	(0.216)	(0.012)	(0.017)
The number of yearly medical malpractice lawsuits		0.002		0.000^c^		0.002^a^		0.000^c^
		(0.001)		(0.000)		(0.001)		(0.000)
Constant	− 15.752^a^	− 9.803	0.549^a^	0.407	− 6.905^a^	0.029	− 0.101	0.114
	(3.868)	(10.691)	(0.152)	(0.255)	(2.278)	(3.656)	(0.120)	(0.178)
Number of observations	9874	8156	9874	8156	13,631	11,311	13,631	11,311
Number of facilities	1958	1882	1958	1882	2909	2814	2909	2814
R-squared (overall)	0.094	0.096	0.059	0.072	0.038	0.049	0.008	0.015

^a, b, c^Represent statistical significance at the 1, 5, and 10 percent levels, respectively. Clustering robust standard errors allowing for correlated residuals within prefectures are in parentheses

As for the clinic sample, increases in the number of physicians and of sickbeds significantly increase both the number of c-section delivery and its ratio. The dummy variable of opening on holidays is also significantly positive, which indicates that c-section delivery and its ratio in those clinics are 1.4 births and approximately 7% higher than otherwise. Unlike with the hospital result, the public dummy is negatively significant, which indicates that the c-section rate in public clinics is 2.7% lower than other hospitals. Regarding environmental variables, the coefficient of the youth population is significantly positive, but its effect is small, as is the case for the results of the hospital sample. In addition, an increase in the number of medical malpractice lawsuits is significantly increase the number of c-section delivery and its ratio, but these effects are also small. Moreover, as with the results for the hospital sample, an increase in the amount of the lump-sum birth allowance significantly increases c-section delivery but that of the density of obstetricians and gynecologists significantly decreases c-section delivery.

### Discussion

This study uses individual medical facility panel data to comprehensively examine the effects of recent public and social environment changes on c-section delivery choice. The empirical results from a fixed effect model show that c-section delivery and the delivery ratio are higher in public hospitals, in relatively large clinics, and in clinics opening on holidays. In addition, increases in the amount of the lump-sum birth allowance and the number of medical malpractice lawsuits also increase the number of c-section deliveries. These effects indicate that an agency problem and defensive medicine exist in Japan, which may increase the average delivery cost.

The empirical results suggest that in Japan, c-section delivery is chosen both in the case of medically justified deliveries, such as high-risk pregnant women, and in the unnecessary case of the supplier’s financial incentive and the social interest of avoiding medical malpractice lawsuits. New guidelines or a new institutional design to control and moderate delivery costs are required in order to minimize unnecessary c-section deliveries.

## Limitations

Our study has some limitations. Although I use longitudinal data at the medical facility level, I do not use data on the pregnant women and infants. To address this problem, I use aggregated proxy variables, but it is unclear how well these variables capture detailed effects. This issue needs to be examined further by using data with more detailed information on mothers, infants, and medical suppliers.

## Abbreviations

c-section: caesarean section
the MHLW: the Ministry of Health, Labour and Welfare
GDP: gross domestic product

## Footnotes

1 In Japan, insurers reimburse medical facilities for the cost of physician services rather than issuing payments directly to individual physicians. In fact, about 70% of doctors in Japan work in medical facilities and they earn a fixed salary irrespective of their workload and outcomes. Thus, physicians serving in medical facilities are not independent economic agents, but instead they function as production inputs for the facilities. The remaining physicians, comprising about 30% of doctors, manage their own clinics, and their income depends on how they provide medical services to patients. Therefore, these practitioners have a greater financial incentive than salaried physicians.

2 Under the *Japanese Medical Care Act*, the definition of a hospital is a medical facility with 20 or more hospital beds. Therefore, some pregnant women deliver in a clinic, which is defined as a medical facility with 19 or fewer hospital beds.

3 In Japan, prefectural governments formulate and conduct their own local health care policy.
